# Investigation of an influenza A(H_1_N_1_)pdm09 outbreak in a high-mobility border district, southwestern Uganda

**DOI:** 10.1016/j.ijregi.2026.100990

**Published:** 2026-09-02

**Authors:** Daniel Wenani, Richard Migisha, Charity Mutesi, Hannington Katumba, Martha Nakaye, Caroline Musubika, Steven Ndugwa Kabwama, Lilian Bulage, Benon Kwesiga, Alex Riolexus Ario

**Affiliations:** Uganda Public Health Fellowship Program, Uganda National Institute of Public Health, Kampala, Uganda

**Keywords:** Influenza A virus, H_1_N_1_ subtype, Respiratory tract infections, Epidemiology, Disease outbreaks, Uganda

## Abstract

**Objectives:**

We investigated to confirm an influenza A(H_1_N_1_)pdm09 outbreak, determine its magnitude, identify factors associated with severe illness, and inform public health response measures in Kisoro District, a high-mobility cross-border district in Uganda.

**Methods:**

We investigated suspected cases from December 1, 2024 to April 4, 2025 using record review and case interviews. Suspected cases had fever with runny nose, cough, or sore throat for ≤10 days plus ≥1 additional symptom or clinician-diagnosed respiratory infection. Severe acute respiratory infection (SARI) was defined as hospitalization or respiratory illness with difficulty breathing. Nasopharyngeal swabs were tested using reverse transcriptase polymerase chain reaction. Factors associated with SARI were assessed using multivariable logistic regression.

**Results:**

We identified 3464 suspected cases; 27% (9/33) of human samples tested positive for influenza A(H_1_N_1_)pdm09. Among suspected cases, the attack rate was highest among children aged <1 year (239/15,845; 15/1000) and those ≥60 years (179/13,365; 13/1000). School-aged children (1-17 years) accounted for 50% of all cases. SARI accounted for 6% (200/3464) of cases. SARI was associated with age 1-17 years (adjusted odds ratio (AOR) = 2.6, 95% confidence interval (CI) = 1.0-6.7) and chronic conditions (AOR = 52, 95% CI = 5-579). Urban residence (AOR = 0.21, 95% CI = 0.1-0.4) and being a pupil or student (AOR = 0.08, 95% CI = 0.02-0.17) were protective.

**Conclusion:**

We confirmed a widespread influenza A(H_1_N_1_)pdm09 outbreak, with predominant transmission among school-going children and greater severity among persons with chronic conditions. Strengthening targeted surveillance, expanded respiratory testing, and school-focused health education are critical to mitigate future outbreaks, particularly in rural and high-mobility cross-border settings.

## Highlights

• Influenza A(H_1_N_1_)pdm09 caused a widespread outbreak in Kisoro, Uganda.
• School-aged children accounted for half of all suspected cases.
• Infants and adults ≥60 years had the highest attack rates.
• Chronic illness was strongly associated with severe disease.
• Border mobility likely amplified outbreak spread and response challenges.

## Background

Influenza A(H_1_N_1_)pdm09, commonly known as swine flu, is an acute respiratory illness caused by an influenza H_1_N_1_ virus subtype that affects humans, swine, and birds. The virus has evolved into new subtypes like the 2009 pandemic type with the potential to cause outbreaks, including pandemics. Swine are primary reservoirs, while humans are secondary reservoirs for H_1_N_1_. H_1_N_1_ can be spread through respiratory droplets, direct contact, and contaminated surfaces [1]. The incubation period for H_1_N_1_ ranges from 1 to 4 days, with an average of 2 days [1]. Children, the elderly, and immunocompromised individuals may shed the virus longer and remain contagious for more extended periods [1].

Influenza A(H_1_N_1_)pdm09 can lead to severe morbidity and even mortality in patients, with many cases requiring hospitalization and, in the most severe instances, intensive care. Influenza poses a significant threat to public health both nationally and globally due to its unpredictable and rapid mutational capabilities [[2], [3], [4], [5], [6], [7]]. This is evidenced by the recent emergence of zoonotic strains including influenza A(H_1_N_1_) [5,6,8]. The 2009 H_1_N_1_ influenza pandemic had a significant global impact, infecting an estimated 0.7-1.4 billion people worldwide within the first year. The World Health Organization reported 575,400 laboratory-confirmed deaths. The United States alone recorded between 8870 and 18,300 deaths, with 43-89 million cases and over 195,000 hospitalizations [9].

In Uganda, the first influenza A(H_1_N_1_)pdm09 case was reported on June 30, 2009, in a businessman returning from England who developed influenza-like illness (ILI) symptoms [7]. Following this introduction, the virus rapidly became the dominant circulating subtype in Kampala, with the epidemic peaking in October 2009. Surveillance data from 2009 to 2015 indicated multiple introductions of the virus in the country, leading to sustained transmission. The 2009 H_1_N_1_ outbreak affected several regions, with school-based outbreaks reporting attack rates as high as 47.1-89.1 per 100 individuals [7]. Although the mortality rate was relatively low, the strain on healthcare resources, increased hospital admissions, and absenteeism from work and school significantly affected the country's economy and social systems.

On March 17, 2025, the district health office notified the Ministry of Health (MoH) of a rise in ILI cases at health facility X in Kisoro District. In response, the national rapid response team was deployed on March 25, 2025. We investigated to confirm, assess the magnitude, and identify factors associated with severity of the outbreak in Kisoro District, a high-mobility border district, to inform control measures.

## Methods

### Outbreak setting

Kisoro District is located in southwestern Uganda, bordering the Republic of Rwanda to the south, the Democratic Republic of Congo (DRC) to the west, Kanungu District to the north, and Rubanda District to the east (Figure 1). It is part of the Kigezi sub-region and lies within the Albertine Rift, a region with rugged terrain and volcanic mountains. The district covers an area of approximately 729 km² and is characterized by steep hills, valleys, and crater lakes. Kisoro is home to part of the Mgahinga Gorilla National Park, which is part of the larger Virunga Conservation Area shared with Rwanda and DRC. The district has a temperate climate influenced by its high altitude, which ranges between 1200 meters and 4127 meters above sea level. It experiences two rainy seasons (March-May and September-November) and two dry seasons (June-August and December-February). The average annual rainfall ranges from 900 mm to 1500 mm, with temperatures ranging between 10°C and 23°C.

**Figure 1:**
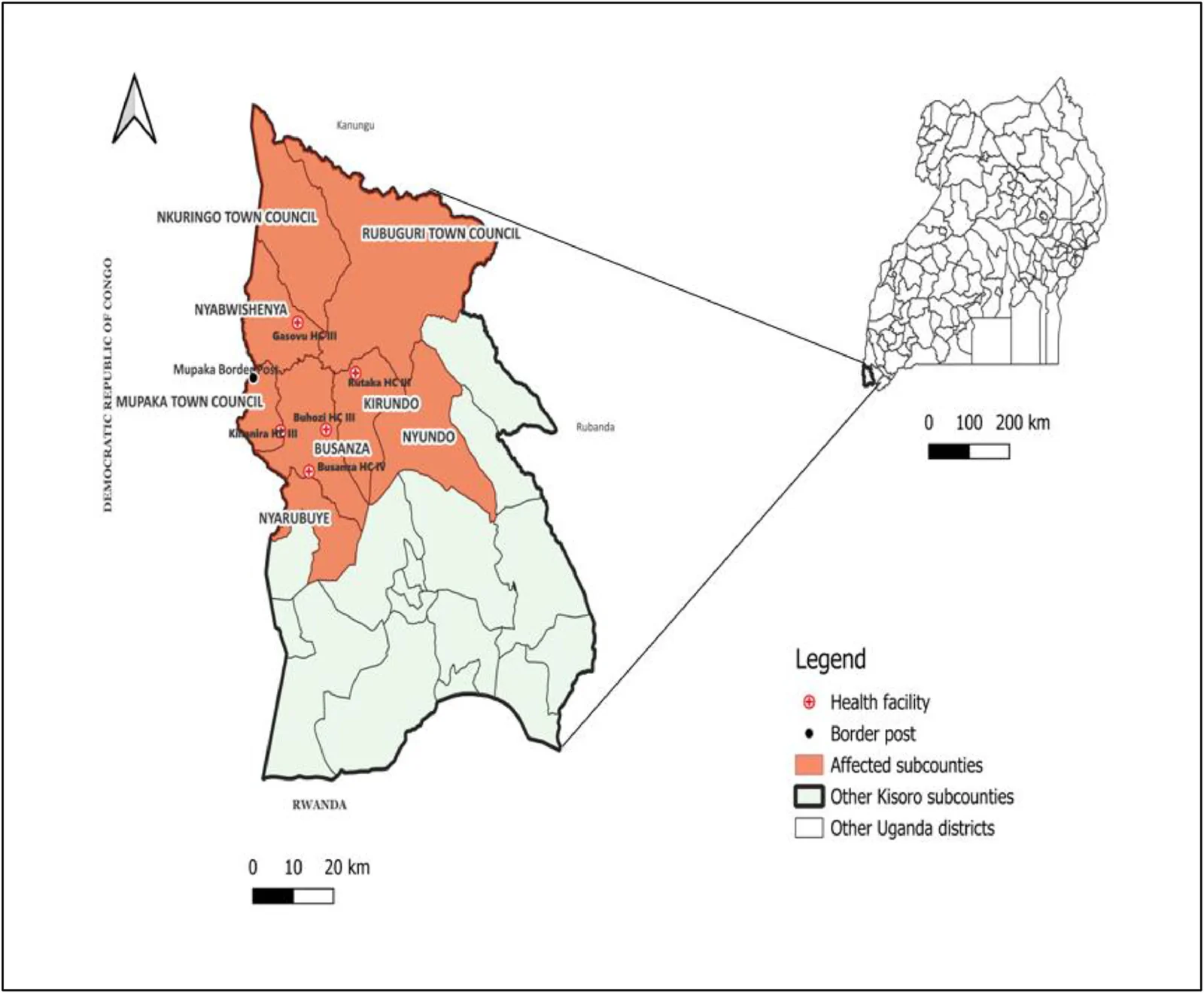
Location of subcounties affected by the influenza A(H_1_N_1_)pdm09 outbreak in Kisoro District, southwestern Uganda, December 2024-April 2025.

According to the 2025 estimates by the Uganda Bureau of Statistics (UBOS), Kisoro District had a population of approximately 342,700 people [10]**.** The district has a high population density, largely due to its limited arable land and dependence on subsistence agriculture. Kisoro’s strategic border location with Rwanda and DRC makes it a high-risk area for the cross-border transmission of infectious diseases, including H_1_N_1_ influenza. The district also has high human mobility due to cross-border trade and migration between Uganda, Rwanda, and DRC, tourism activities, and refugee settlements. The district has 24 administrative units, including subcounties and town councils. In Uganda, influenza surveillance includes routine facility-based reporting of respiratory illness through the national surveillance Health Management Information System and sentinel ILI/ severe acute respiratory infection (SARI) surveillance at selected health facilities. Sentinel surveillance includes collection and laboratory testing of respiratory specimens for influenza and other respiratory viruses.

### Case definitions

#### Suspected and confirmed H_1_N_1_ case

We defined a suspected H_1_N_1_ case as the acute onset of fever with runny nose or cough or sore throat lasting ≤10 days with at least one of the following symptoms: difficulty breathing, headache, lethargy, myalgia, joint pain, diarrhea, vomiting, and convulsions with or without hospitalization, or a clinician's diagnosis suggestive of acute respiratory infection, Kisoro District, December 1, 2024-April 4, 2025. We defined a confirmed H_1_N_1_ case as a suspected case with laboratory confirmation of the H_1_N_1_ pathogen in Kisoro District from December 1, 2024-April 4, 2025.

#### ILI and SARI

We incorporated case definitions for ILI and SARI. We defined an ILI case as an acute onset of fever with runny nose or cough or sore throat without difficulty breathing, or a clinician’s diagnosis of acute respiratory infection not requiring hospitalization. A SARI case was defined as acute onset of fever with cough, runny nose or sore throat, accompanied by difficulty breathing or requiring hospitalization.

#### Case finding

We conducted case finding by reviewing medical records primarily at healthcare facilities that had reported an increase in the number of acute respiratory infections in Kisoro District with diagnoses suggestive of H_1_N_1_ influenza or ILI/SARI. Additionally, we interviewed active cases in healthcare facilities prospectively using a case investigation form. Systematic household-based community case finding was not conducted. The case investigation form consisted of information on demographic characteristics, clinical data such as the time of symptom onset and duration, social history, any laboratory investigations done, treatment given, potential factors associated with SARI, and outcomes. All cases were line-listed. Given the overlapping clinical presentation of respiratory infections, other respiratory illnesses were considered in the differential diagnosis, and case finding therefore included patients with ILI, SARI, and other clinician-diagnosed acute respiratory infections, while influenza A(H_1_N_1_)pdm09 remained the primary pathogen under investigation.

#### Descriptive epidemiology

We characterized cases by person, place, and time. Person characteristics included sex, age, and occupation. We categorized age (in years) into four meaningful categories: <1 year, 1-17 years, 18-59 years, and ≥60 years. The place characteristics included the distribution of suspected case patients by subcounty (attack rates) and village and demonstration by use of Quantum Geographic Information System. We described cases by time using an epidemic curve. Additionally, we profiled suspected H_1_N_1_ cases by their presenting signs and symptoms. We reviewed District Health Information software version 2 DHIS2 data to check for seasonality and estimate the burden of the current influenza A(H_1_N_1_)pdm09 outbreak in the district. We further described SARI cases by person, place, and time, and assessed factors associated with severity of illness.

#### Assessment of factors associated with SARI

We conducted a cross-sectional study nested within the H_1_N_1_ outbreak investigation. Data used for this analysis were obtained from the H_1_N_1_ line list compiled during the outbreak investigation. The dataset included demographic, clinical, and epidemiological variables such as age, sex, residence, occupation, presence of chronic illness, and pregnancy status. The dependent variable was the presence of SARI. Independent variables included age group (<1 year, 1-17 years, 18-59 years, and ≥60 years), sex, residence (urban/rural), occupation, having a chronic condition, and pregnancy status. We conducted a bivariable analysis using a chi-square test to calculate crude odds ratios (COR) with 95% confidence intervals (CIs) using STATA^TM^ version 16. Variables at *P* < 0.05 were considered statistically significant and were subsequently included in a multivariable logistic regression model to estimate adjusted odds ratios (AORs) with 95% CIs.

#### Laboratory investigations

At each reporting health facility, all suspected case patients eligible for specimen collection were identified and selected using simple random sampling. The human nasopharyngeal specimens were tested by reverse transcriptase polymerase chain reaction for influenza viruses and severe acute respiratory syndrome coronavirus 2 (SARS-CoV-2). Following negative SARS-CoV-2 results and detection of influenza viruses, subsequent laboratory and epidemiological investigations focused on influenza, particularly influenza A(H_1_N_1_)pdm09. Two animal nasopharyngeal swabs were collected from swine in the village with the highest number of cases. All human samples and one animal sample were transported to the Uganda Virus Research Institute for testing, while the other animal sample was sent to the Central Public Health Laboratory via the hub transport system.

#### Environmental assessment

We conducted key informant interviews with health workers, the District Health Team, the District Veterinary Officer, and local and opinion leaders to identify the likely source of the outbreak. We visited schools in the most affected village to assess infection prevention and control (IPC) measures and to engage the school administrators on the status of the pupils with ILIs. We also assessed IPC measures at Mupaka Border Post and evaluated porosity of the border.

## Results

### Confirming the existence of influenza A(H_1_N_1_)pdm09 outbreak, Kisoro District, Uganda, December 2024-April 2025

#### Trends of ILI in Kisoro District, 2020-2025

Following the analysis of the DHIS2 data, no seasonal pattern of influenza-like illnesses was noted from 2020 to 2025. A marked increase in reported ILI cases was observed in September 2024, followed by a decline. In 2025, the number of ILI cases reported through DHIS2 was lower than the number identified from facility registers during the outbreak investigation (Figure 2).

**Figure 2:**
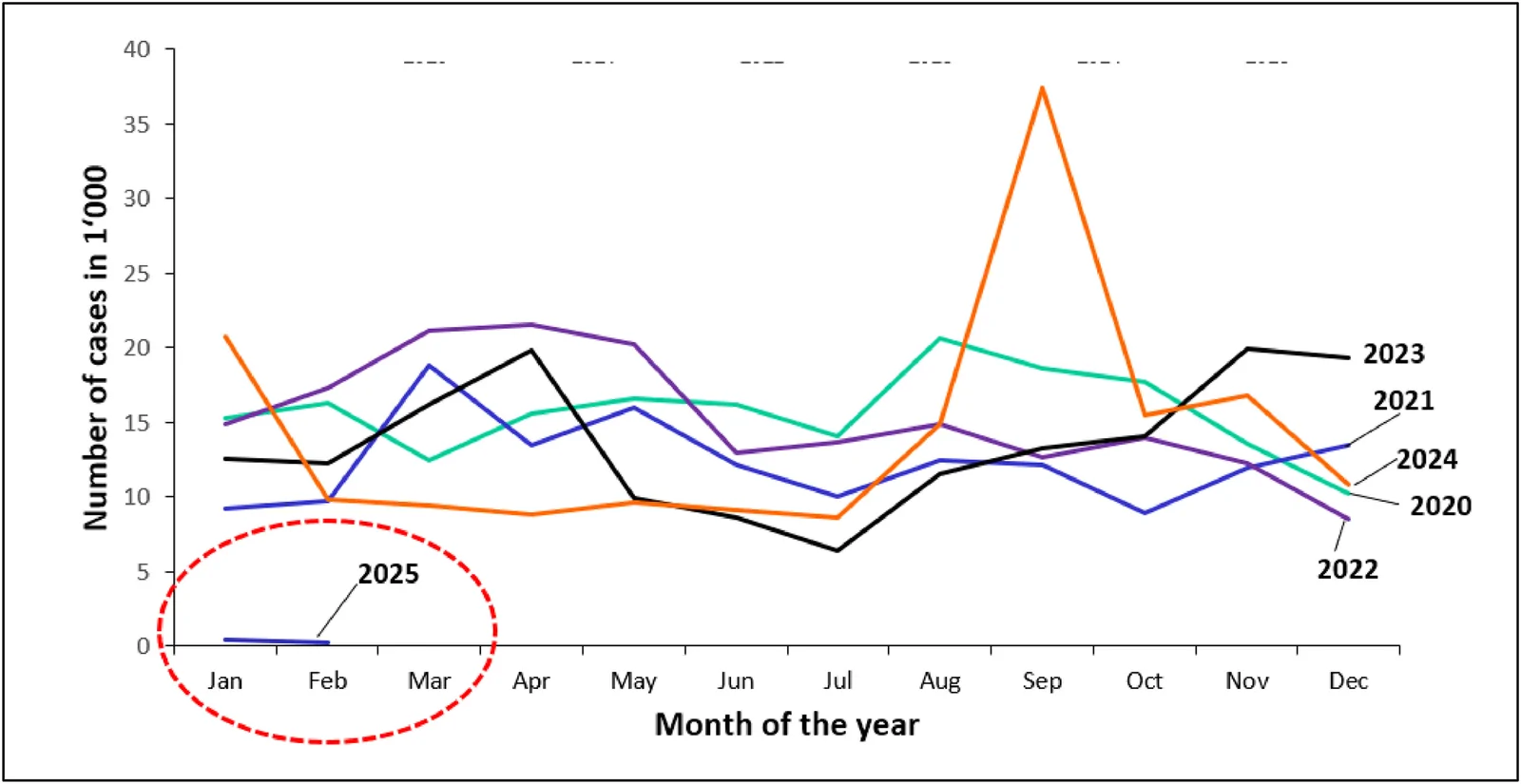
Temporal trends in reported ILI cases in Kisoro District, Uganda, 2020-2025, using routine (DHIS2) surveillance data.

#### Laboratory investigation

Of the 33 human samples collected, nine (27%) tested positive for influenza A(H_1_N_1_)pdm09. In addition, two (6%) samples were positive for influenza A but could not be subtyped, and one (3%) was positive for influenza B (Victoria lineage), giving an overall influenza positivity of 12/33 (36%). Two animal samples from swine from the village that had the highest number of cases returned negative for any influenza viruses.

### Descriptive epidemiology of suspected influenza A(H_1_N_1_)pdm09 cases, Kisoro District, December 2024-April 2025

#### Person and clinical characteristics of case patients during influenza A(H_1_N_1_)pdm09 outbreak investigation, Kisoro District, Uganda, December 2024-April 2025

A total of 3464 suspected cases were identified from December 2024 to April 4, 2025. The highest proportions of these cases were identified at Buhozi health care (HC) III [n = 1370 (39.5%)] and Busanza HC IV [n = 1117 (32.2%)]. Other cases were identified at Gasovu HC III [n = 396 (11.4%)], Rutaka HC III [n = 385 (11.1%)], and Kinanira HC III [n = 196 (5.6%)].

Females [n = 2080 (60%)] had a higher proportion of cases. Those aged 1-17 years had the highest proportion of cases with 1751 (50%). Eight hundred ninety-nine (26%) cases were aged 18-59 years, 239 (7%) were those aged <1 year, and 179 (5%) were aged ≥60 years.

Females (AR = 11/1000 population) were more affected than males (AR = 9/1000 population). Among the age categories, the <1-year age group (AR = 15/1000) and the ≥60-year age group (AR = 13/1000) had the highest attack rates (Table 1).

**Table 1:** Sociodemographic and clinical characteristics of suspected influenza A(H_1_N_1_)pdm09 cases, Kisoro District, December 2024-April 2025.

Variable	Number of cases, *n*	(%)	Population	AR/1000
**Sex**				
Female	2080	(60)	188,600	11
Male	1384	(40)	154,100	9
**Age group (years)**				
<1	239	(7)	15,845	15
1-17	1751	(50)	156,190	11
18-59	899	(26)	157,299	6
≥60	179	(5)	13,365	13
**Occupation**				
Peasant	414	(12)		
Preschool children	198	(6)		
Pupil or student	366	(10)		
Others (carpenter, teachers, builder, tailor)	16	(1)		
Unknown	2470	(71)		
**Diagnoses**				
RTI	1392	(40)		
Cold or cough (no pneumonia)	756	(22)		
Common cold	401	(12)		
Coryza	381	(11)		
Pharyngitis or tonsillitis or sore throat	197	(6)		
Pneumonia	167	(5)		
SARS^a^	62	(2)		
Severe pneumonia	38	(1)		
Bronchitis or bronchiolitis	37	(1)		
Rhinitis	33	(1)		
**Symptoms (*n* = 770)**				
Fever	770	(100)		
Cough	585	(76)		
Runny nose	578	(75)		
Headache	231	(30)		
Chills	162	(21)		
Chest pain	139	(18)		
Sore throat	139	(18)		
Difficult breathing	31	(4)		
Diarrhea	8	(1)		
**Hospitalization status**				
Yes	200	(6)		
No	3264	(94)		
**Had chronic condition**				
Yes	18	(1)		
No	3446	(99)		
**Female and pregnant**				
Yes	33	(1)		
No	2047	(59)		
Missing values	1384	(40)		

AR, attack rate; RTI, respiratory tract infection; SARS, severe acute respiratory syndrome.

a SARS diagnosis was documented in the register and was not independently verified during the investigation.

Peasants accounted for 414 (12%) of the cases, pupils or students 366 (10%), and preschool children 198 (6%), while the occupations of most cases were not documented.

One thousand three hundred ninety-two (40%) cases had respiratory tract infection (RTI) and 756 (22%) cases had cold or cough (no pneumonia) as diagnoses. Four hundred one (12%) had common cold, 381 (11%) coryza, 197 (6%) pharyngitis or tonsillitis or sore throat, 167 (5%) pneumonia, 62 (2%) SARS, 38 (1%) severe pneumonia, 37 (1%) bronchitis or bronchiolitis, and 33 (1%) rhinitis. Apart from fever, cases presented with respiratory symptoms such as cough and runny nose. Two hundred (6%) of the cases were hospitalized. Eighteen (1%) had chronic conditions. Thirty-three (1%) were female and pregnant.

#### Distribution of influenza A(H_1_N_1_)pdm09 cases over time in Kisoro District, December 2024-April 2025

There was a first wave of cases in early December 2024 with cases mainly from Busanza Subcounty. There were cases from DRC in the first wave in early December 2024 (Figure 3). The number of cases in the first wave decreased toward the end of January 2025. The second wave of cases started in early February 2025 and peaked in mid-March 2025. The outbreak investigation was initiated on March 28, 2025 when cases were reducing in number.

**Figure 3:**
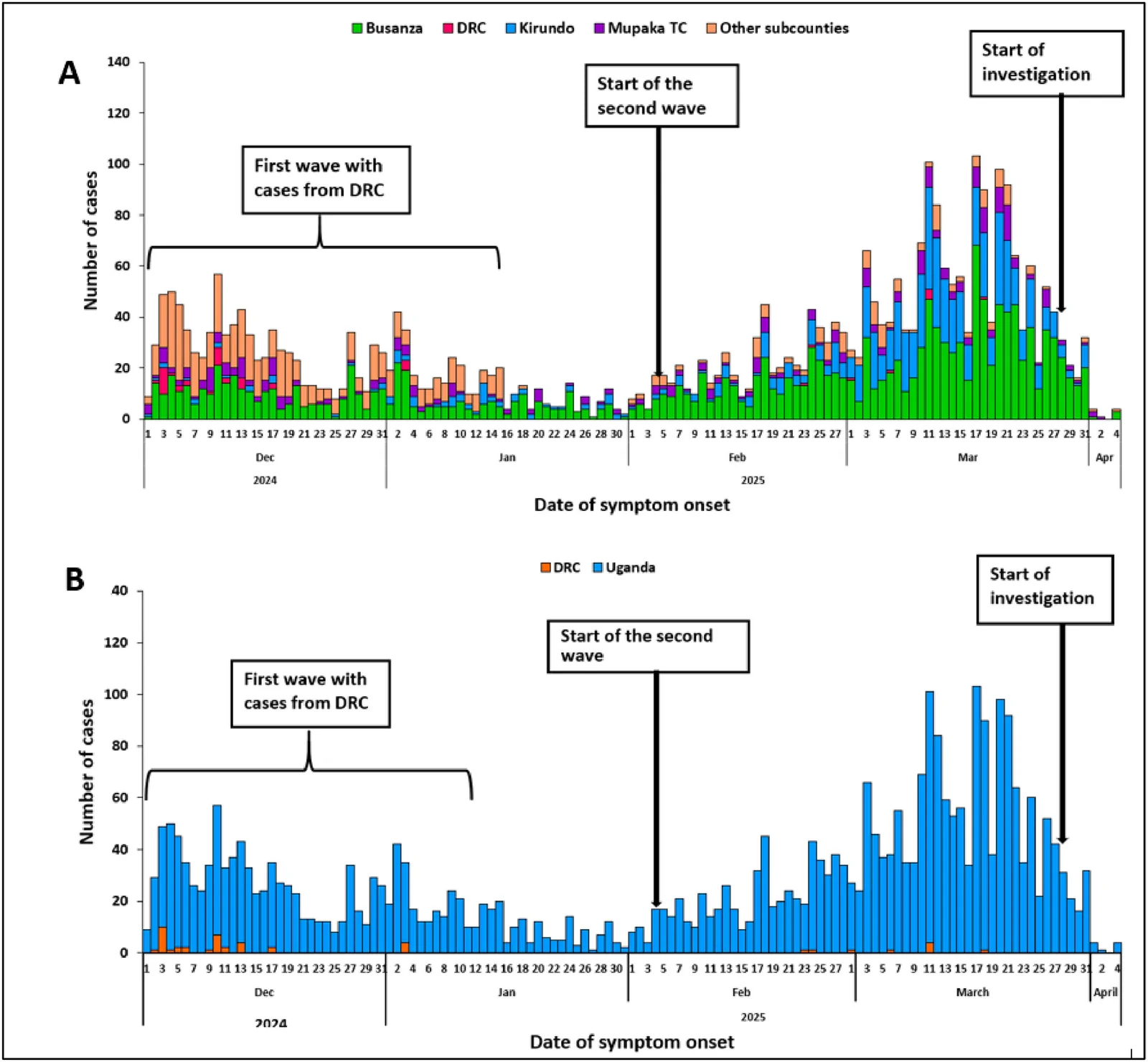
Distribution of suspected influenza A(H_1_N_1_)pdm09 cases over time (A, stratified by subcounty, and B, stratified by epidemiological linkage to Democratic Republic of Congo vs Uganda) in Kisoro District, Uganda, December 2024-April 4, 2025 (n = 3464).

#### Distribution of suspected influenza A(H_1_N_1_)pdm09 cases by subcounty during the H_1_N_1_ outbreak, Kisoro District, December 2024-April 2025

Eight subcounties were affected, i.e. Rubuguri Town Council (TC), Kirundo SC, Busanza SC, Nyundo SC, Nkuringo TC, Nyarubuye SC, Mupaka TC, and Nyabwishenya SC. Busanza and Kirundo SC were the most affected subcounties (Figure 4).

**Figure 4:**
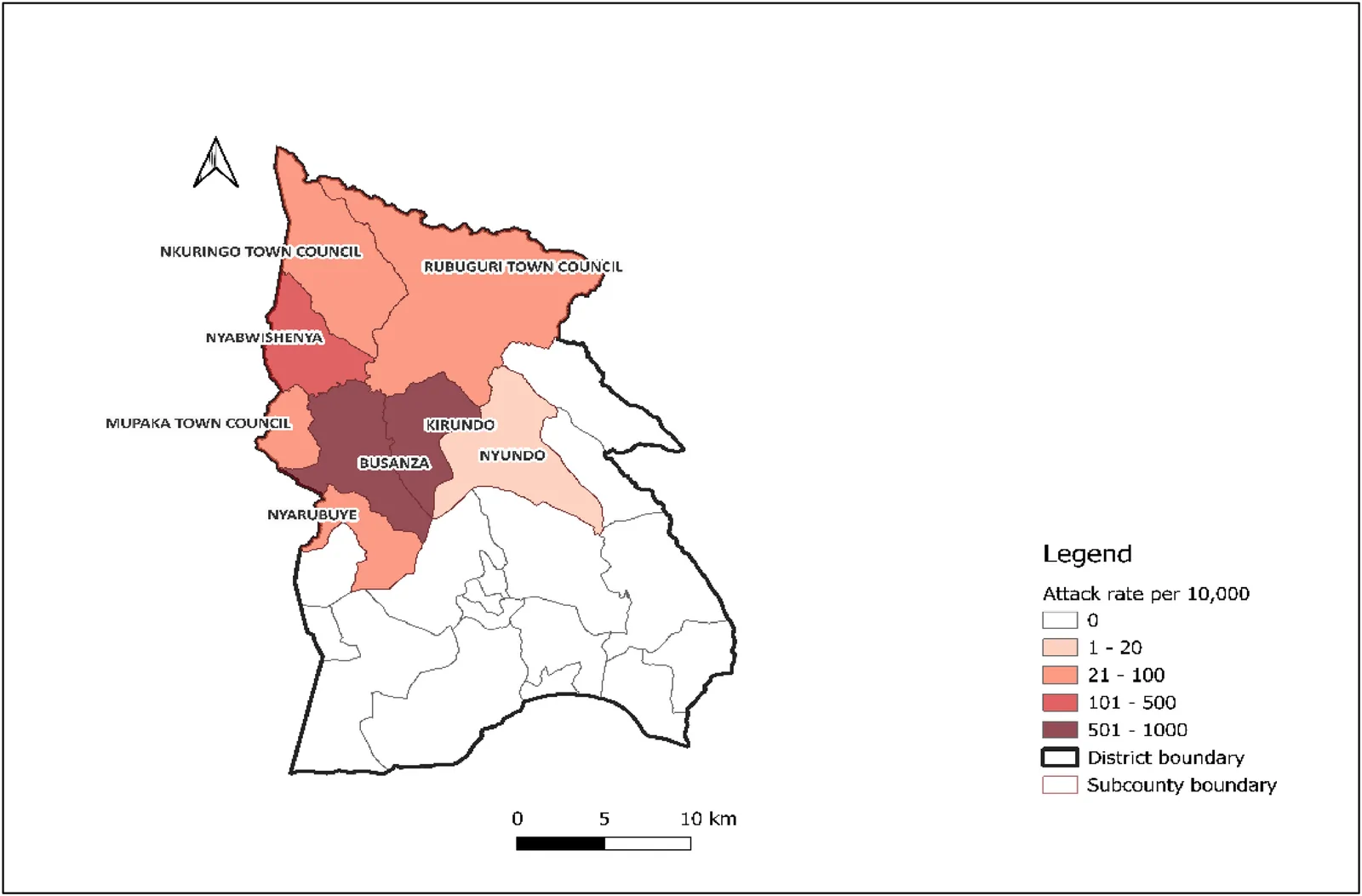
Suspected influenza A(H_1_N_1_)pdm09 attack rates by subcounty, Kisoro District, Uganda, December 2024-April 2025.

#### Descriptive epidemiology of the SARI cases

There were 200 patients with SARI. The majority of the SARI cases were identified at Rutaka HC III (117; 59%) and Buhozi HC III (61; 31%). Other cases were from Kinanira HC III (19; 10%) and Busanza HC IV (2; 1%). Females (AR = 7/10,000) had a higher attack rate than males (AR = 5/10,000) (Table 2). Those aged ≥60 years (AR = 17/10,000) and those aged <1 year (AR = 13/10,000) were the most affected. RTI, pneumonia, severe pneumonia, and SARS were the most common diagnoses. Six patients had comorbidities, and four were pregnant (Table 3).

**Table 2:** Distribution of SARI cases during the H_1_N_1_ outbreak, Kisoro District, March-April 2025.

Variable	Cases, *n*	(%)	Population	AR/10,000
**Health facility**				
Rutaka HC III	117	(59)		
Buhozi HC III	61	(31)		
Kinanira HC III	19	(10)		
Busanza HC IV	2	(1)		
Gasovu HC III	1	(1)		
**Sex**				
Female	129	(65)	188,600	7
Male	71	(36)	154,100	5
**Age group (years)**				
<1	21	(11)	15,845	13
1-17	90	(45)	156,190	6
18-59	66	(33)	157,299	4
≥60	23	(12)	13,365	17

AR, attack rate; HC, Health Center; SARI, severe acute respiratory infection.

**Table 3:** Common respiratory pathologies associated with SARI during the H1N1 outbreak, Kisoro District, March-April 2025.

Variable	Cases, n	(%)
**Diagnoses**		
RTI	107	(54)
Pneumonia	51	(26)
Severe pneumonia	17	(9)
SARS	16	(8)
Bronchitis or bronchiolitis	3	(2)
Cold or cough (no pneumonia)	2	(1)
Common cold	2	(1)
Coryza	1	(1)
Pharyngitis or tonsillitis or sore throat	1	(1)
**Other conditions**		
Comorbidity	6	
Pregnancy	4	

RTI, respiratory tract infection; SARI, severe acute respiratory infection; SARS, severe acute respiratory syndrome.

### Factors associated with SARI

In bivariate analysis, being aged ≥60 years (COR = 1.9, 95% CI = 1.1-3.1, P = 0.02) and having a chronic condition (COR = 11, 95% CI = 4-28, P < 0.001) were significantly associated with SARI (Table 4). Age group 1-17 years compared with the 18-59-year age group was associated with lower odds of SARI (COR = 0.67, 95% CI = 0.5-0.9, P = 0.02). Pupils or students were less likely to develop SARI than peasants (COR = 0.23, 95% CI = 0.14-0.4, P < 0.001). Urban residents had reduced odds of developing SARI relative to rural residents (COR = 0.58, 95% CI = 0.34-1.0, P = 0.048). Sex and pregnancy were not statistically associated with SARI.

**Table 4:** Bivariable and multivariable analysis of factors associated with severe acute respiratory infection during the influenza A(H_1_N_1_)pdm09 outbreak, Kisoro District, Uganda, December 2024-April 2025.

Variable	SARI cases		ILI cases		COR	(95% CI)	P-value	AOR	(95% CI)	P-value
	n	(%)	n	(%)						
**Sex**										
Male	71	(35)	1313	(40)	Ref					
Female	129	(65)	1951	(60)	1.2	(0.9-1.6)	0.18		-	
**Age group (years) (n = 3067)**										
<1	17	(9)	222	(8)	0.97	(0.6-1.7)	0.9	2.5	(0.8-8.1)	0.12
1-17	88	(45)	1662	(58)	0.67	(0.5-0.9)	**0.02**	2.6	(1.0-6.7)	**0.04**
18-59	66	(34)	833	(29)	Ref			Ref		
≥60	23	(12)	156	(5)	1.9	(1.1-3.1)	**0.02**	1	(0.5-2.0)	0.95
**Residence**										
Rural	185	(92)	2867	(88)	Ref			Ref		
Urban	15	(8)	397	(12)	0.58	(0.34-1)	**0.048**	0.21	(0.1-0.4)	**<0.001**
**Occupation (n = 994)**										
Peasant	75	(54)	339	(40)	Ref			Ref		
Pupil or student	18	(13)	348	(41)	0.23	(0.14-0.4)	**<0.001**	0.08	(0.02-0.17)	**<0.001**
Preschool child	43	(31)	155	(18)	1.2	(0.82-1.9)	0.2	0.6	(0.18-1.3)	0.263
Others^a^	3	(2)	13	(1)	1	(0.29-3.8)	0.95	1.3	(0.34-4.9)	0.72
**Had chronic condition**^b^										
No	193	(97)	3253	(99)	Ref			Ref		
Yes	7	(3)	11	(1)	11	(4-28)	**<0.001**	52	(5-579)	**0.001**
**Pregnant**										
No	125	(97)	1922	(98)	Ref					
Yes	4	(3)	29	(2)	2.1	(0.8-6.5)	0.13			

AOR, adjusted odds ratio at multivariable analysis; CI, confidence interval; COR, crude odds ratio at bivariable analysis; ILI, influenza-like illness; SARI, severe acute respiratory infection.

a Others included teachers, carpenters, and tailors

b Chronic conditions were heart disease, *e.g.,* hypertension, heart failure; chronic lung disease, *e.g.,* asthma, chronic obstructive pulmonary disease; chronic kidney disease; chronic liver disease; diabetes; sickle cell disease; cancer; HIV; and tuberculosis. Missing values were excluded from the analysis.

In multivariable analysis, individuals aged 1-17 years had higher odds of SARI than those in other age groups (AOR = 2.6, 95% CI = 1.0-6.7). Those with a chronic condition had increased odds of SARI (AOR = 52, 95% CI = 5-579) (Table 4). Conversely, urban residence (AOR = 0.21, 95% CI = 0.1-0.4) and being a pupil or student (AOR = 0.08, 95% CI = 0.02-0.17) were associated with reduced odds of SARI.

#### Environmental assessment

Key informants reported animal deaths, particularly among swine, beginning in September 2024 in Busanza and Kirundo subcounties, with similar reports from other subcounties across the district. They reported that a sick swine was sold at the home of a confirmed case around March 2025.

Observation at Mupaka Border Post (near the DRC border) showed that IPC measures were not strictly implemented. In addition, insecurity in eastern DRC related to the M23 conflict limited cross-border coordination and access, preventing joint investigation and verification with DRC public health authorities [11]. Frequent cross-border movement through porous routes, including travel by DRC nationals into Uganda, was reported and may have increased importation risk and complicated follow-up of suspected cases.

At Pre and Primary School X, the school administration reported an increase in pupils presenting with ILI about 2 weeks prior, with many seeking care at Buhozi HC III. The rise was reported mainly among pupils in Primary Three and Primary Four classes.

## Discussion

This investigation was conducted in response to an increase in acute respiratory illness and confirmed an outbreak of influenza A(H_1_N_1_)pdm09 in Kisoro District, southwestern Uganda, characterized by widespread community transmission and a high burden among school-aged children. The highest attack rates were observed among infants aged <1 year and adults aged ≥60 years, with females disproportionately affected. The outbreak unfolded in two distinct waves, beginning in early December 2024 and again in early February 2025, with a peak in mid-March 2025. Spatially, transmission was concentrated in eight subcounties, particularly Kirundo and Busanza, which are located near the border with the Democratic Republic of Congo. In the analytical component, age 1-17 years and the presence of chronic conditions were associated with higher odds of SARI, whereas urban residence and being a pupil or student were associated with lower odds of SARI.

The outbreak was concentrated in subcounties near the Mupaka Border Post along the Democratic DRC border, suggesting that cross-border population movement may have contributed to early spread. This risk is likely heightened by porous borders and limited infection prevention and control (IPC) measures at border crossing points [12], [13]. Similar cross-border transmission patterns of influenza A(H_1_N_1_)pdm09 have been reported in other settings, highlighting the importance of coordinated cross-border surveillance and response [12], [14]. Ongoing conflict in eastern DRC may have further weakened routine influenza surveillance and reporting, making timely detection and containment of cross-border cases more challenging [14], [15]. Clinically, fever, cough, and runny nose were the most commonly reported symptoms, consistent with typical influenza presentation; however, many cases were initially classified as non-specific respiratory tract infections or other syndromic diagnoses. This misclassification points to gaps in case recognition at peripheral health facilities and underscores the need to strengthen the use of standardized case definitions and diagnostic practices.

The age-specific attack rates show that infants younger than 1 year and adults aged 60 years and older were more affected, which is consistent with previous studies showing that influenza tends to be more severe in people with weaker immune responses at the extremes of age [5], [8], [16], [17], [18], [19]. School-aged children accounted for most cases, likely because close contact in school settings makes it easier for influenza to spread, a pattern commonly observed in other influenza outbreaks [[20], [21], [22]]. These findings highlight the importance of school-based surveillance and early control measures to detect outbreaks quickly and limit further transmission in such high-risk areas. Children aged 1-17 years had approximately threefold higher odds of developing SARI. This group includes young children under 5 years, who are known to experience more severe influenza, potentially due to age-related immune responses or delays in care-seeking among older children within this age group [19,23]. Although infants under 1 year also showed increased odds of severity, the association missed statistical significance.

The presence of an underlying chronic condition was the strongest predictor of severe disease, with a markedly elevated adjusted odds of SARI. Among the SARI cases, the documented chronic conditions were predominantly hypertension and chronic respiratory diseases, including asthma. The strong association between chronic conditions and SARI emphasizes the vulnerability of individuals with underlying illnesses [24]. Additionally, patients with cardiopulmonary chronic conditions have reduced physiologic reserve and can have exacerbated hypoxemia and lower respiratory tract complications during influenza infection, increasing the likelihood of hospitalization [25,26]. These populations need to be prioritized for surveillance, clinical follow-up, and preventive interventions.

Urban residence was significantly protective, possibly due to earlier healthcare access, better health-seeking behavior, or reduced environmental risk factors 25. Conversely, rural residents may face barriers such as delayed presentation, transportation challenges, or limited health facility capacity [21]. The reduced odds observed among pupils and students, compared to peasants, may be attributed to earlier detection through school-based surveillance, better health status, or age-related physiological resilience [27], [28]. This is consistent with findings that although school-aged children are highly exposed, they often present with less severe disease [28].

The epidemic curve demonstrated two temporal peaks in suspected respiratory illness. However, laboratory confirmation of influenza A(H_1_N_1_)pdm09 was available only during the later period of the investigation. Consequently, the December 2024-January 2025 peak cannot be conclusively attributed to influenza A(H_1_N_1_)pdm09 and may have represented a separate respiratory illness event. The available data are therefore insufficient to determine whether the two peaks were part of a single continuous outbreak.

Overall, the findings from this investigation highlight the need to strengthen early warning surveillance systems for influenza, particularly in rural and cross-border districts with high population mobility. Integrating facility-based SARI surveillance with school-based and community event-based surveillance could improve early detection and response. The higher odds of severe illness among infants, older adults, and individuals with underlying conditions highlight the need to prioritize these groups for targeted prevention, early care-seeking, and timely referral. Improving district-level diagnostic capacity and strengthening referral systems are critical to reduce delays in diagnosis and treatment. Together, these measures could enhance influenza preparedness, reduce severe outcomes, and improve outbreak response in high-risk and resource-limited settings.

## Limitations

This investigation had limitations. First, the reliance on retrospective data collection from facility registers introduced the potential for underreporting and misclassification due to inconsistent documentation, particularly where DHIS2 data did not align with paper-based records. This likely masked the true burden and trend of influenza-like illnesses at the onset of the outbreak. SARS diagnosis documentation in the registers was not independently clinically verified and misclassification might have been possible. Second, there was a high proportion of missing data, especially on variables such as occupation and age, which limited the ability to fully assess risk factors across all strata. These gaps may have biased the multivariable regression analysis or reduced the statistical power to detect true associations. Third, laboratory confirmation was limited by testing capacity and logistics during the response. Although a large number of suspected cases were identified (n = 3464), only 33 human specimens were collected and tested. This small and potentially non-representative subset limits inference about overall positivity, subtype distribution, and the extent of co-circulating respiratory pathogens, and may have led to overestimation of the true contribution of influenza A(H_1_N_1_)pdm09 to the outbreak. Similarly, only a few animal specimens were collected, limiting our ability to assess animal-to-human transmission; moreover, even detection of influenza in animals would not conclusively establish direction of transmission. Systematic household-based case finding was not conducted and therefore, persons with mild illness who did not seek healthcare at the health facilities and other unreported cases in the community may have been missed. The use of census population denominators with facility-detected cases may also underestimate the true population incidence and may be influenced by differences in healthcare-seeking behavior. The reported case rates should consequently not be interpreted as true population attack rates. Finally, insecurity in eastern DRC constrained cross-border coordination and access, preventing joint investigation and verification with DRC public health authorities [11].

## Conclusion

This investigation confirmed that influenza A(H_1_N_1_)pdm09 caused a widespread community outbreak in Kisoro District, with severe illness significantly associated with the presence of chronic conditions. Infants and older adults experienced the highest attack rates, while pupils and students and urban residents had reduced odds of severe disease. The findings demonstrate the continued public health threat posed by influenza in high-mobility border settings and underscore the importance of early detection and timely clinical care. Strengthening routine surveillance and outbreak preparedness remains critical for preventing severe outcomes during future influenza epidemics.

## Public health actions

We conducted health education at primary schools, where the highest number of cases was reported. We educated pupils on proper hand hygiene and isolation practices. We also conducted health education at the health facilities and initiated the practice of patients wearing masks to prevent the spread of infection. We supported the district in having its first district task force meeting where the findings of the investigation were disseminated.

## Funding

The project was supported by the President’s Emergency Plan for AIDS Relief (PEPFAR) through the United States Centers for Disease Control and Prevention Cooperative Agreement number GH001353-01 through Makerere University School of Public Health to the Uganda Public Health Fellowship Program, Ministry of Health. The findings and conclusions in this report are those of the authors and do not necessarily represent the official views of the US Centers for Disease Control and Prevention, the Department of Health and Human Services, the Agency for Toxic Substances and Disease Registry, the Makerere University School of Public Health, or the Uganda Ministry of Health.

## Ethical approval

This outbreak investigation was in response to a public health emergency and was therefore determined to be non-research. MoH gave permission to investigate this outbreak. In agreement with the International Guidelines for Ethical Review of Epidemiological Studies by the Council for International Organizations of Medical Sciences (1991) and the Office of the Associate Director for Science, US CDC/Uganda, it was determined that this activity was not human subjects research and that its primary intent was public health practice or disease control activity (specifically, epidemic or endemic disease control activity). This activity was reviewed by the US CDC and was conducted consistent with applicable federal law and CDC policy. See, *e.g.,* 45 C.F.R. part 46, 21 C.F.R. part 56; 42 U.S.C. §241(d); 5 U.S.C. §552a; 44 U.S.C. §3501 et seq. Prior to data collection, informed verbal consent was obtained from all the participants who were 18 years or older (legal age in Uganda). For those below 18 years, consent was sought from their parents/guardians and assent was also obtained from them to participate in the study. Since the investigation was conducted during an outbreak of an acute respiratory infection, verbal consent was used to minimize prolonged close contact and unnecessary handling and exchange of shared materials, such as paper consent forms and pens.

## Consent for publication

Not applicable.

## Availability of data and materials

The datasets on which our findings are based belong to the Uganda Public Health Fellowship Program and MoH. For confidentiality reasons, the datasets are not publicly available. However, the datasets are available upon reasonable request from the corresponding author with permission from the Uganda Public Health Fellowship Program and MoH.

## CRediT authorship contribution statement

**Daniel Wenani:** Conceptualization, Data curation, Formal analysis, Investigation, Methodology, Project administration, Software, Supervision, Validation, Visualization, Writing – original draft. **Richard Migisha:** Conceptualization, Investigation, Supervision, Validation, Writing – review & editing. **Charity Mutesi:** Conceptualization, Data curation, Investigation, Writing – review & editing. **Hannington Katumba:** Conceptualization, Investigation, Writing – review & editing. **Martha Nakaye:** Investigation, Methodology. **Caroline Musubika:** Investigation, Methodology. **Steven Ndugwa Kabwama:** Writing – review & editing. **Lilian Bulage:** Writing – review & editing. **Benon Kwesiga:** Writing – review & editing. **Alex Riolexus Ario:** Funding acquisition, Resources, Writing – review & editing.

## Declaration of competing interest

The authors have no competing interests to declare.
